# Imaging and clinical efficacy analysis of minimally invasive reduction and crossbar external fixation in the treatment of Sanders Ⅱ and Ⅲ calcaneal fractures

**DOI:** 10.3389/fsurg.2025.1550024

**Published:** 2025-05-07

**Authors:** Jianchuan Wang, Tianmin Guan, Qiwen Xue

**Affiliations:** ^1^ School of Mechanical Engineering, Dalian Jiaotong University, Dalian, China; ^2^ Department of Orthopedics, Affiliated Zhongshan Hospital of Dalian University, Dalian, China

**Keywords:** calcaneus fracture, external fixation needle, lateral L-shaped approach, percutaneous reduction, sinus tarsi approach

## Abstract

**Objective:**

To evaluate the clinical efficacy of percutaneous minimally invasive reduction combined with crossbar external fixation in the treatment of Sanders type II and III calcaneal fractures.

**Methods:**

A retrospective analysis was performed on 65 patients with Sanders type II and III calcaneal fractures who were treated at Zhongshan Hospital affiliated with Dalian University between February 2019 and June 2021. Among them, 48 were males and 17 were females, with a mean age of 42.3 ± 10.2 years. There were 45 cases of Sanders type II fractures and 20 cases of type III fractures. Patients were divided into three groups based on the surgical methods: Group A (*n* = 25, minimally invasive reduction with crossbar external fixation), Group B (*n* = 21, minimally invasive locking plate fixation via the sinus tarsi approach), and Group C (*n* = 19, locking plate fixation via the lateral L-shaped incision). The following parameters were recorded and compared among the groups: preoperative waiting time, operative duration, intraoperative blood loss, incision length, and postoperative complications. Imaging parameters assessed included calcaneal length, width, height, Böhler angle, Gissane angle, and varus angle. Clinical outcomes were evaluated using the American Orthopedic Foot and Ankle Society (AOFAS) ankle-hindfoot score, Visual Analogue Scale (VAS), Short Form Health Survey (SF-36), and Maryland Foot and Ankle Score.

**Results:**

Baseline characteristics showed no significant differences among the groups (*P* > 0.05). All patients were followed up for an average duration of 15.6 ± 1.2 months. Group A demonstrated significantly shorter preoperative waiting times, operative durations, lower intraoperative blood loss, and shorter incision lengths compared with groups B and C (*P* < 0.05). Furthermore, VAS scores 3 days postoperatively were significantly lower in group A compared to groups B and C (*P* < 0.05), whereas no significant difference was observed between groups B and C. The incidence of postoperative complications was significantly lower in groups A and B compared with group C (*P* < 0.05), with no significant difference observed between groups A and B. In addition, complications observed in the study included pinhole infections, cyanosis of the skin at the edge of the incision, nerve damage, and skin necrosis. Statistically, the complication rate was significantly lower in the group with the percutaneous minimally invasive approach than in the group with the traditional L-shaped approach. Imaging assessments at 2 weeks and 12 months postoperatively revealed no significant differences among the three groups in Böhler angle, Gissane angle, calcaneal varus angle, and calcaneal dimensions (*P* > 0.05). All imaging parameters significantly improved postoperatively within each group (*P* < 0.05). At the final follow-up, clinical outcomes (AOFAS, SF-36, Maryland scores) showed no significant differences among groups (*P* > 0.05).

**Conclusion:**

The Percutaneous minimally invasive reduction combined with crossbar external fixation provides effective fixation with minimal trauma, shorter hospital stays, and lower complication rates, representing a viable treatment strategy for Sanders type II and III calcaneal fractures.

## 1 Introduction

Calcaneal fractures are relatively common injuries in clinical practice, representing the most frequent type of tarsal fracture. According to existing literature, calcaneal fractures account for up to 60% of all tarsal fractures and approximately 2% of all fractures system-wide, with nearly 75% classified as displaced intra-articular fractures (1). The Sanders classification system—originally based on coronal CT imaging of the posterior facet—remains pivotal for surgical decision-making, particularly in distinguishing Type II (two-part intra-articular) from Type III (comminuted central depression) fractures, as highlighted in recent historical analyses of calcaneal fracture management (2). Although modern surgical techniques have dramatically improved the prognosis of fractures, nonoperative treatment still plays an important role in specific clinical contexts and in case selection. Early conservative strategies focused on cast bracing and closed manipulation, which avoided the risk of invasive surgery but was limited by the inability to accurately restore the anatomical alignment of the subtalar joint surfaces, resulting in residual unevenness of the joint surfaces and abnormal heel morphology, which may lead to a higher risk of long-term traumatic arthritis. Conservative treatment is still considered a viable option, especially for patients with severe soft tissue injuries or general conditions that make surgery unfavorable. The limitations of these options have prompted the academic community to explore minimally invasive procedures that are more compatible with the need for anatomic reconstruction. With the emergence of the concept of accelerated rehabilitation, optimization of the surgical approach is essential to promote early recovery. The Sanders classification is based on semi-coronal CT scans at the widest segment of the posterior articular facet. Three fracture lines (A/B/C) were hypothesized: line C corresponds to the medial edge of the talar articular surface, while lines A (lateral) and B (medial) divide the lateral articular facet into equal thirds. Type II fractures involve a two-part split of the posterior facet (subtypes IIA, IIB, or IIC), whereas type III fractures exhibit central depression with three main fragments (subtypes IIIAB, IIIAC, or IIIBC), depending on the dominant fracture line orientation. These injuries typically occur due to vertical impacts sustained from falls, causing substantial disruption to calcaneal anatomy. Conservative treatment often results in an increased risk of developing long-term traumatic arthritis. Although surgical intervention for displaced calcaneal fractures is widely accepted, there remains controversy regarding the optimal surgical approach. With the emergence of accelerated rehabilitation protocols, optimizing surgical methods has become critically important for facilitating early patient recovery.

Currently, open reduction and internal fixation (ORIF) represent the primary methods for managing calcaneal fractures, with the lateral extended “L"-shaped incision widely adopted. However, incision-related complications remain a significant concern, with soft tissue complication rates ranging from 20% to 37% (3, 4), causing apprehension among surgeons. The sinus tarsi approach, a minimally invasive technique, has gained popularity due to reduced soft tissue complications but still has limitations, including restricted exposure, difficulty accessing the fracture site, and potential risk of sural nerve injury (5). Studies have shown that various types of plates, screws, and intramedullary fixation devices can provide sufficient biomechanical stability for fractures (6). No significant advantage of locked plates was found in the treatment of calcaneal fractures. Additionally, traditional internal fixation with metal plates or screws often necessitates secondary operations for implant removal after fracture healing, increasing the risks and costs associated with further trauma and interventions.

With advances in minimally invasive orthopedic surgical techniques, their application in treating calcaneal fractures has progressively expanded. Therefore, identifying effective minimally invasive methods has become a key focus in clinical research to avoid the above-mentioned complications (7). External fixation avoids secondary incisions and can be conveniently removed in an outpatient setting, meeting patient needs effectively.

Guided by minimally invasive principles, this study retrospectively analyzed the clinical outcomes of Sanders type II and III calcaneal fractures treated with percutaneous minimally invasive reduction combined with longitudinal and transverse interwoven external fixation at the Department of Trauma and Orthopedics, Zhongshan Hospital Affiliated to Dalian University from February 2019 to June 2021. The results were compared with those treated using minimally invasive locking plate fixation via the sinus tarsi approach and traditional L-shaped lateral incision within the same period, aiming to provide alternative methods and insights for managing intra-articular calcaneal fractures.

## 2 Materials and methods

### 2.1 General information

This study was conducted as a retrospective cohort analysis. Sixty-five patients diagnosed with Sanders type II and III calcaneal fractures, treated surgically at our hospital between February 2019 and June 2021, were included according to specific inclusion criteria. Patients were divided into three groups based on the surgical approach used: Group A underwent minimally invasive reduction with crossbar external fixation; Group B was treated with minimally invasive locking plate fixation via the sinus tarsi approach; and Group C received traditional locking plate fixation via the lateral L-shaped incision. The gender imbalance (male:female = 1,149:557) reflects the occupational exposure pattern of calcaneal fractures in our region (8).

Group A comprised 25 patients (18 males, 7 females) with an average age of 43.2 ± 9.7 years (range: 25–62 years), including 13 right-sided and 12 left-sided fractures. Injury mechanisms included falls from height (*n* = 20) and traffic accidents (*n* = 5). Fractures were classified as Sanders type II in 14 cases and type III in 11 cases.

Group B consisted of 21 patients (15 males, 6 females) with an average age of 41.5 ± 10.7 years (range: 25–64 years), including 12 right-sided and 9 left-sided fractures. Causes of injury were falls from height (*n* = 13) and traffic accidents (*n* = 8). Fracture classifications included Sanders type II (*n* = 12) and type III (*n* = 9).

Group C included 19 patients (12 males, 7 females) with an average age of 42.4 ± 8.7 years (range: 26–65 years), comprising 11 right-sided and 8 left-sided fractures. The mechanisms of injury included falls from height (*n* = 12) and traffic accidents (*n* = 7). Fracture classifications were Sanders type II in 10 cases and type III in 9 cases.

Baseline characteristics for the three groups are summarized in Table 1.

**Table 1:** Comparison of general conditions among the three groups.

Group	Sex（case）		Age (year,`x ± s)	Sidelong (case)		Sanders type		Injury mechanism	
	male/female			Left/right		Ⅱ	Ⅲ	Fall**/**Traffic injuries	
A group	18	7	43.2 ± 9.7	12	13	14	11	20	5
B group	15	6	41.5 ± 10.7	9	12	12	9	13	8
C group	12	7	42.4 ± 8.7	8	11	10	9	12	9
Test statistics	5.425		0.685	1.211		1.608		0.364	
*P*-value	0.452		0.342	0.523		0.651		0.576	

Note: Group A was treated with percutaneous minimally invasive reduction and crossbar interwoven external fixation needle. Group B was treated with minimally invasive locking plate for tarsal sinus incision. Group C was treated with L-shaped incision locking plate.

### 2.2 Preoperative preparation

After admission, the affected limb was elevated, immobilized with plaster, treated with ice application, and managed with anti-swelling measures. Lateral-axis radiographs, lateral x-rays, and three-dimensional CT scans of the calcaneus were obtained. Preoperative Böhler angle, Gissane angle, calcaneal varus angle, calcaneal length, width, and height were measured and recorded. The patients were not randomized or blinded; instead, a detailed explanation of the advantages and disadvantages of each surgical method was provided to the patients and their families preoperatively, allowing them to choose their preferred method. Patients in Group A underwent immediate surgery after completing imaging examinations, without waiting for swelling to subside. Patients in Groups B and C underwent surgery once the “skin striation” sign appeared on the lateral aspect of the foot. All surgical procedures were performed by the same senior surgeon and team.

### 2.3 Surgical method

To mitigate selection bias, consecutive cases meeting inclusion criteria from 2019.2 to 2021.6 were enrolled. Preoperative variables (e.g., Böhler angle, Gissane angle, calcaneal varus angle, calcaneal length, width, and height) were analyzed as potential predictors via multivariate regression. Patients underwent surgery under spinal or general anesthesia, positioned laterally. Group A underwent closed surgery, while groups B and C were operated under tourniquet control.

**Figure 1:**
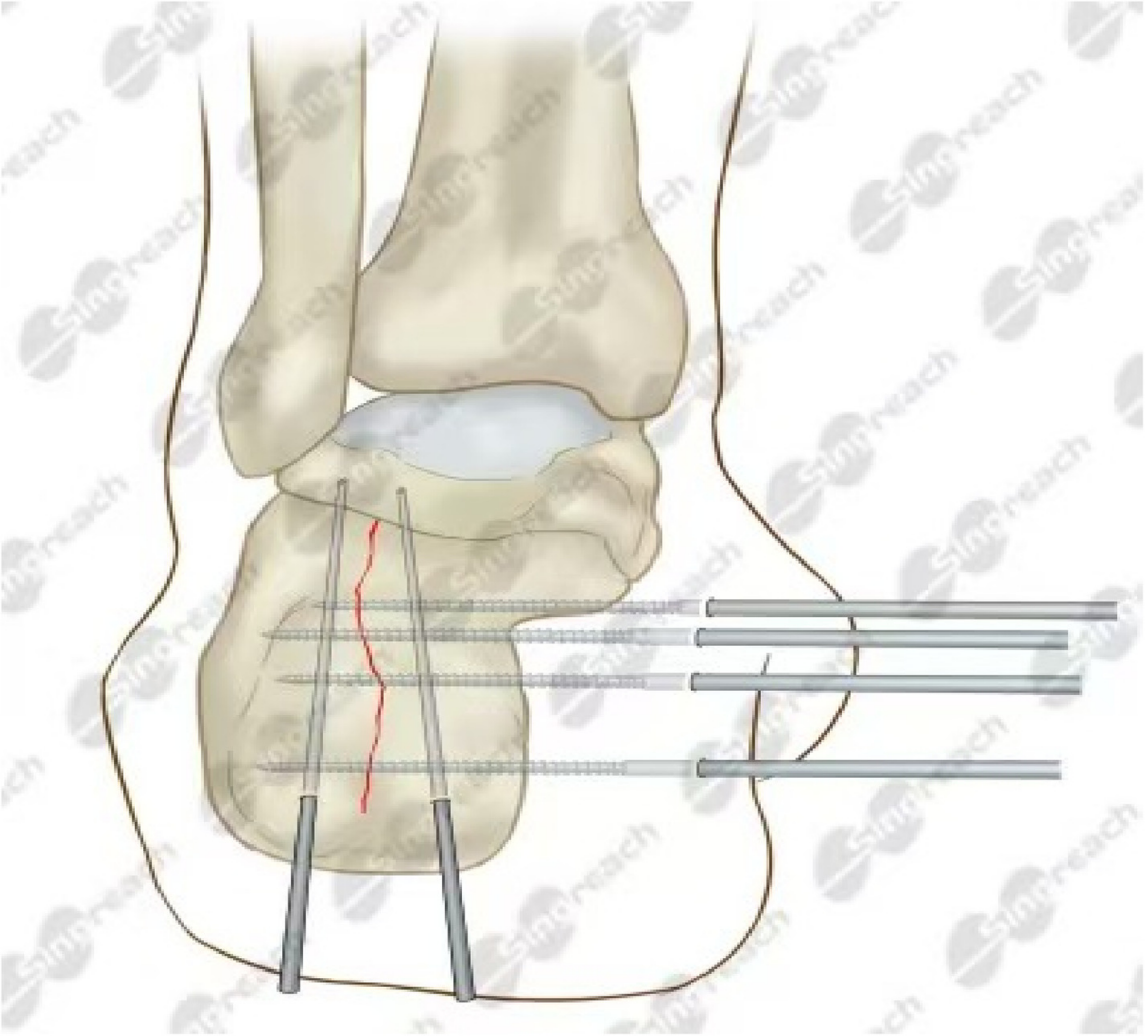
Percutaneous minimally invasive reduction and external fixation of a Sanders type II/III calcaneal fracture with transverse bar. Under C-arm fluoroscopic guidance, a Kirschner wire is first inserted. Traction repositioning is then performed to correct the shortening, flexion deformity, and width of the calcaneus. The collapsed articular surfaces were elevated to restore the Burrer and Giese angles. Two threaded pins support the posterior articular surface to control the deformity. Three additional pins reinforce the fixation of the calf bone dimensions. Finally, external fixation pins were attached in a triangular pattern and the ankle was immobilized in a cast.

**Figure 2:**
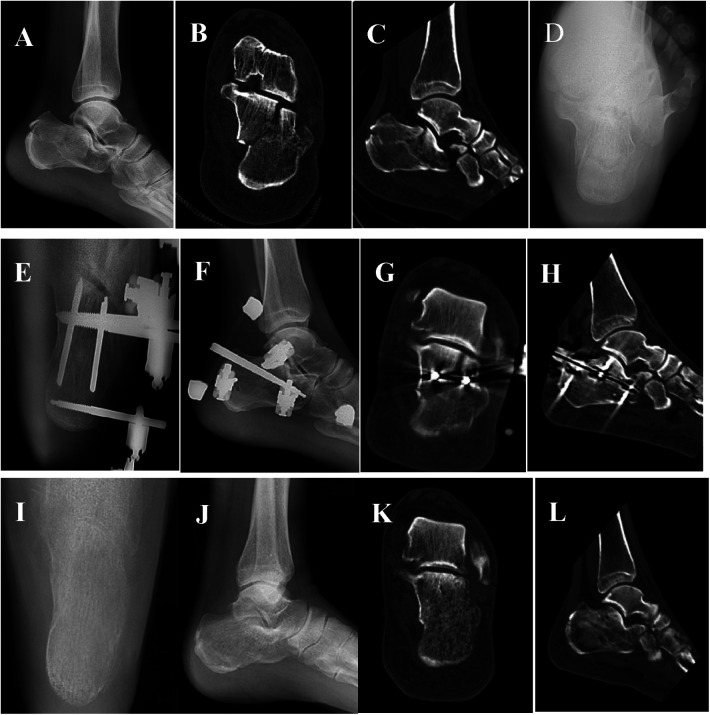
A 45-year-old man with a left calcaneal comminuted fracture caused by a fall, sanders III AB type, was subjected to percutaneous minimally invasive reduction with calcaneal reduction forceps and crossbar external fixation. He was followed up for 12.8 months. **(A,B)** Axial and lateral x-ray images showed calcaneus shortening, widening of width, decreasing of height, and varus deformity. **(C,D)** coronal and sagittal plain CT scan showed subtalar posterior articular split and collapse. **(E,F)** Two thread pins were fixed parallel to the anterior articular surface via the medial and lateral walls of the calcaneus respectively at the x-ra*y* axis after operation to control the length of the calcaneus and varus; The lateral view shows that two thread needles are fixed closely to the articular surface from the back up to the front down to hold up the collapsed articular surface, and the other three thread needles are fixed to a surface at three points, which can further control the length, width and height of the calcaneus. **(G,H)** Coronal and sagittal CT images showed that the posterior articular surface below the talar was flat, and Bohler Angle and Gissane Angle returned to the normal Angle range. **(I,J)** From the last follow-up, axial and lateral x-ray images showed good recovery in length, width, and height. **(K,L)** CT showed that the subtalar posterior articular surface was stable.

**Figure 3:**
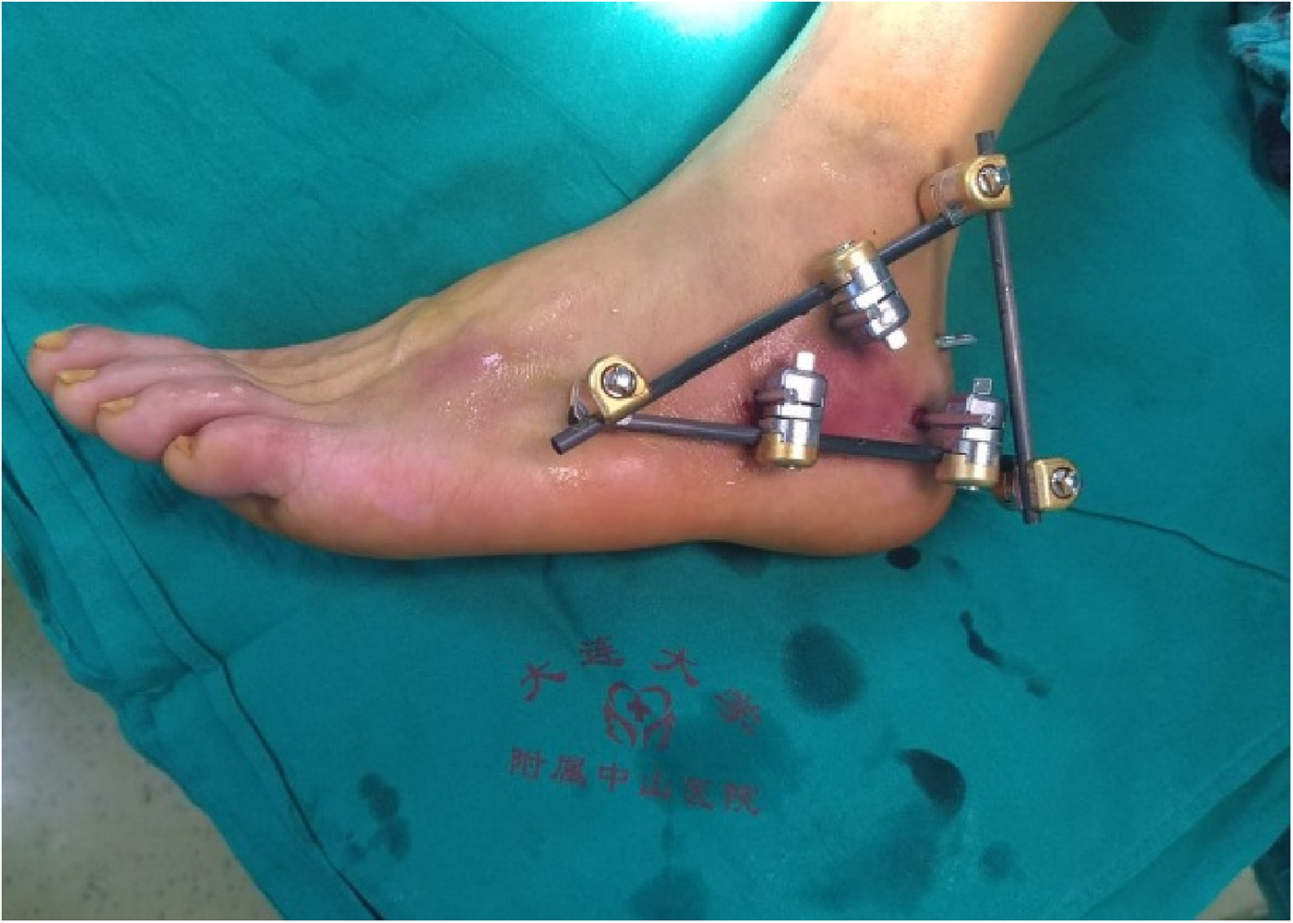
External observation after percutaneous minimally invasive reduction with calcaneal reduction forceps and crossbar external fixation.

Group A: Under C-arm fluoroscopy, a 4.0 mm Kirschner wire was inserted from the medial to the lateral aspect of the posterior calcaneus. Traction reduction was performed using calcaneal reduction forceps, assisted by counter-traction between the calf and forefoot. Initially, the shortening of the calcaneus was corrected to restore length; then the varus deformity was corrected, and calcaneal width was restored through manual compression. The collapsed articular surface was elevated under continuous monitoring by C-arm fluoroscopy until Böhler and Gissane angles returned to normal ranges. First, two threaded pins were inserted from the highest point of the posterior calcaneal tuberosity through the Gissane angle toward the calcaneocuboid joint surface. Axially, these two pins were placed close to the medial and lateral cortices to support the elevated posterior articular surface, maintain calcaneal length, and control varus deformity. Next, three additional pins were inserted posteriorly into the calcaneus, positioned respectively at the Gissane angle, posterior articular surface, and inferior to the articular surface, following a three-point fixation principle to reinforce control of calcaneal length, width, and height (Figure 1). Finally, external fixation pins were shortened and interconnected in a triangular pattern using pin clamps and carbon-fiber rods. Postoperatively, the ankle joint was immobilized in a functional position with plaster (Figures 2, 3).

Group B: A sinus tarsi incision approximately 4–6 cm long was made with care to protect the sural nerve. After the incision of the skin, the peroneal tendon was retracted downward, exposing the collapsed articular surface step by step. Limited exposure of the lateral calcaneus body was achieved to facilitate plate insertion. A 4.0 mm Kirschner wire was drilled vertically into the calcaneus to correct its length and varus deformity. The collapsed articular surface was elevated using a periosteal elevator, and lateral-to-medial compression restored calcaneal width and height. A minimally invasive locking plate was then inserted and secured sequentially with locking screws. Proper positioning was confirmed by C-arm fluoroscopy. The incision was closed, drainage was placed, pressure dressing applied, and functional plaster fixation of the ankle joint was performed.

Group C: A standard lateral L-shaped incision was made on the calcaneus, cutting through the skin and subcutaneous layers carefully to protect the sural nerve and peroneal tendon sheath. The skin flap was maintained by suture retraction. The posterior calcaneal articular surface was fully exposed, and the collapsed fragments were elevated using periosteal elevators. Bone grafting was performed at defect sites. Adequate reduction was confirmed using C-arm fluoroscopy, after which a locking plate was placed along the lateral calcaneal wall and fixed with locking screws. A negative-pressure drainage tube was placed, and the incision was closed in layers. Finally, a pressure dressing and functional ankle plaster fixation were applied.

### 2.4 Postoperative treatment

Groups B and C were routinely administered antibiotics for three days postoperatively, along with bemiparin sodium anticoagulation therapy. The affected limbs were elevated to reduce swelling, and patients were instructed to perform functional exercises including toe movements, hip and knee flexion-extension, and quadriceps strengthening. Pin sites in Group A were sterilized regularly with alcohol-soaked cotton swabs, whereas wound areas in Groups B and C were disinfected using alcohol gauze dressings. Incisions were kept dry, and drainage was monitored closely. Drainage tubes were removed 24–48 h postoperatively, depending on drainage output.

Follow-up imaging with x-ray and CT scans was routinely conducted at 1, 2, 4, 8, 12 weeks, and again at 6 and 12 months postoperatively. Sutures and ankle plaster immobilization were removed two weeks after surgery. For Group A, the external fixation device was removed in the outpatient setting at 6–8 weeks postoperatively. All patients commenced partial weight-bearing ambulation (25% of body weight) using crutches at eight weeks, gradually increasing weight-bearing by approximately 25% weekly, and reaching full weight-bearing at 12 weeks postoperatively.

### 2.5 Clinical and imaging observation indicators

Clinical evaluation: Preoperative waiting time, operative duration, intraoperative blood loss, incision length, and postoperative Visual Analog Scale (VAS) pain scores at 3 days were recorded for patients in all three groups (9). At the final follow-up, clinical outcomes were assessed using the American Orthopaedic Foot and Ankle Society (AOFAS) Ankle-Hindfoot Score (10), the Short Form-36 (SF-36) Health Survey (11), and the Maryland Foot and Ankle Score (12).

Imaging evaluation: Measurements were performed independently by two experienced radiologists. Calcaneal length, width, height, Böhler angle, Gissane angle, and calcaneal varus angle were measured and compared preoperatively, at 2 weeks, and at 12 months postoperatively to assess the quality of fracture reduction.

Postoperative complications: Incidence of incision infection, skin necrosis, subcutaneous hematoma, nerve injury, traumatic arthritis, and other complications were recorded and compared among the three groups.

### 2.6 Statistical analysis

An independent biostatistician blinded to group allocation performed all analyses using SPSS v26.0 software. The Shapiro–Wilk test was performed first to assess the normality of continuous data. Continuous data conforming to normal distribution and homogeneity of variance were presented as mean ± standard deviation (x¯ ± s). Comparisons among the three groups were performed using one-way analysis of variance (ANOVA), and pairwise comparisons were conducted using paired *t*-tests. Categorical data were analyzed using the chi-square test. A *P*-value of less than 0.05 was considered statistically significant.

## 3 Results

### 3.1 General information

Baseline characteristics showed no statistically significant differences among the three groups (*P* > 0.05). All patients completed follow-up, with an average duration of 15.6 ± 1.2 months (range: 13–25 months).

### 3.2 Clinical outcome

Group A exhibited significantly shorter preoperative waiting times, operative durations, incision lengths, and lower intraoperative blood loss compared with Groups B and C (all *P* < 0.05). Pairwise comparisons between groups were also statistically significant (*P* < 0.05). The postoperative VAS scores at 3 days in Group A were significantly lower than in Groups B and C (*P* < 0.05), whereas no significant difference was observed between Groups B and C (*P* > 0.05, Table 2).

**Table 2:** Comparison of clinical results among the three groups.

Group	Waiting time (d)	Operation time (min)	Peroperative bleeding (ml)	Incision length (cm)	VAS score 3 days after surgery
A group	2.6 ± 1.2	65.5 ± 10.9	20.1 ± 15.2	0.4 ± 0.1	2（3,4）
B group	7.2 ± 1.9	90.6 ± 8.5	65.3 ± 20.6	5.0 ± 0.8	7（6,8）
C group	8.0 ± 2.8	98.5 ± 12.5	83.7 ± 18.1	12.5 ± 1.2	9（8,10）
*F*-value	8.324	6.216	3.253	203.457	223.466
*P*-value	<0.001	<0.001	<0.001	<0.001	<0.001

Note: Group A was treated with percutaneous minimally invasive reduction and crossbar interwoven external fixation needle. Group B was treated with the minimally invasive locking plate for the tarsal sinus incision. Group C was treated with L-shaped incision-locking plate. The comparison between group a and group A was <0.05, and the comparison between group b and group B was <0.05.

At the final follow-up, no significant differences were found among the three groups regarding AOFAS and SF-36 scores (*P* > 0.05). Maryland Foot and Ankle Scores indicated excellent results in 21 patients, good in 2, and fair in 2 (92% excellent-to-good rate) for Group A; 18 excellent, 1 good, and 2 fair (90.5%) for Group B; and 14 excellent, 3 good, and 2 fair (89.5%) for Group C. No significant differences in Maryland scores were identified among groups (*P* > 0.05, Table 3).

**Table 3:** Clinical function scores of patients in the 3 groups at the last follow-up.

Group	AOFAS score	SF-36 score	Maryland score
A group	91.2 ± 3.4	80.9 ± 3.2	90.2 ± 3.8
B group	89.8 ± 5.5	79.3 ± 4.7	88.7 ± 2.6
C group	88.3 ± 1.3	78.2 ± 2.9	87.5 ± 2.5
*F-*value	2.065	1.983	2.957
*P-*value	0.203	0.184	0.115

Note: Group A was treated with percutaneous minimally invasive reduction and crossbar interwoven external fixation needle. Group B was treated with minimally invasive locking plate for the tarsal sinus incision. Group C was treated with L-shaped incision locking plate.

There were statistically significant differences in postoperative complications among the three groups (*P* < 0.05). Group A: only 1 case (4%) developed pinhole infection, which healed successfully after removal of the needle with local dressing change without further complications; Group B: 2 cases (about 9.6%) developed mild cyanosis of the skin at the edge of the incision, which were recovered after local care and dressing change without nerve or deep infection; Group C (lateral incision with L-shaped approach to locking the plate): a total of 4 cases (21.1%) developed complications, including 2 cases of nerve injury and 2 cases of skin necrosis. included 2 cases of nerve injury and 2 cases of skin necrosis. All patients were treated with appropriate neurotrophic therapy and surgical debridement and repair; and ultimately improved. The incidence of postoperative complications in Groups A and B was significantly lower than in Group C (*P* < 0.05), whereas no significant difference was observed between Groups A and B (*P* > 0.05).

### 3.3 Imaging findings

No significant differences among groups were found in radiographic parameters (Böhler angle, Gissane angle, calcaneal varus angle, length, width, and height) at 2 weeks and 12 months postoperatively (*P* > 0.05). All parameters significantly improved postoperatively compared to preoperative values within each group (*P* < 0.05). No significant differences in these parameters were found between the 2-week and 12-month postoperative assessments (*P* > 0.05; Table 4).

**Table 4:** Comparison of imaging indexes of patients in the three groups at the last follow-up.

Group	Bohler angle(°)		*t-*value	*P-value*	Gissane angle(°)		*t-*value	*P-*value
	Pre-operative	Post-operative			Pre-operative	Post-operative		
A group	10.6 ± 1.7	32.5 ± 3.4	8.056	<0.001	95.6 ± 2.3	124.2 ± 9.8	9.876	<0.001
B group	11.8 ± 3.5	30.1 ± 2.8	9.654	<0.001	94.3 ± 3.6	123.4 ± 8.5	9.434	<0.001
C group	11.5 ± 2.3	31.3 ± 1.2	10.231	<0.001	95.1 ± 4.2	122.5 ± 5.2	8.542	<0.001
*F-*value	1.342	1.870			0.957	1.786		
*P-*value	0.438	0.395			0.896	0.367		
Group	Varus angle(°)		*t-*value	*P-*value	Calcaneal length(cm)		*t*-value	*P-*value
	Pre-operative	Post-operative			Pre-operative	Post-operative		
A group	12.3 ± 2.3	4.8 ± 3.1	10.877	<0.001	4.13 ± 1.6	6.35 ± 1.8	3.757	<0.001
B group	12.1 ± 3.8	4.5 ± 2.9	12.106	<0.001	4.03 ± 2.2	6.28 ± 1.5	2.968	<0.001
C group	12.0 ± 4.2	4.3 ± 2.3	11.564	<0.001	4.01 ± 2.7	6.54 ± 1.2	3.132	<0.001
*F* value	1.082	0.279			0.718	1.364		
*P* value	0.196	0.825			0.665	0.299		
Group	Calcaneal width(cm)		*t*-value	*P-*value	Calcaneal height(cm)		*t-*value	*P-*value
	Pre-operative	Pre-operative			Pre-operative	Post-operative		
A group	4.12 ± 1.6	3.35 ± 2.4	3.172	<0.001	4.05 ± 0.8	4.96 ± 1.1	3.879	<0.001
B group	4.43 ± 0.4	3.42 ± 1.8	3.645	<0.001	3.98 ± 1.7	4.85 ± 0.5	3.688	<0.001
C group	4.25 ± 2.1	3.28 ± 1.3	4.254	<0.001	40.1 ± 2.7	4.13 ± 2.2	2.695	<0.001
*F* value	0.745	0.972			0.896	0.946		
*P* value	0.586	0.463			0.513	0.375		

Group A was treated with percutaneous minimally invasive reduction and crossbar interwoven external fixation needle. Group B was treated with minimally invasive locking plate for the tarsal sinus incision. Group C was treated with L-shaped incision locking plate.

## 4 Discussion

The calcaneus is typically an irregular structure composed of a thin layer of cortical bone surrounding rich cancellous bone. When a calcaneal fracture occurs, the irregular shape of the calcaneus deforms, leading to a decrease in its height, which shortens the Achilles tendon lever arm, causes limb length discrepancy, and makes it difficult to wear shoes (13). A decrease in the height of the calcaneus and the Bohler's angle simultaneously can lead to a “horizontal talus,” which results in reduced subtalar joint mobility, navicular subluxation, severe pain, and flatfoot deformity (14). Sanders type II/III fractures pose significant reconstructive challenges due to articular displacement (15). Achieving anatomical correction of Böhler/Gissane angles and subtalar alignment remains critical for functional outcomes (16). These complications primarily arise because the lateral soft tissue of the calcaneus is thin and fragile, making it susceptible to surgical trauma. Additionally, the lateral calcaneal artery, located near the incision corner, may easily be injured during surgery, further impairing soft tissue healing. Despite the extensive approach, inadequate reduction of the posterior facet joint still occurs due to its irregular shape and limited visibility of the medial aspect of the joint surface (17).

With an increased understanding of minimally invasive surgical principles in orthopedic practice, several minimally invasive techniques have been introduced to reduce complications, including external fixation, percutaneous fixation, and minimally invasive approaches such as the sinus tarsi approach. Compared with traditional open surgery, minimally invasive techniques ensure effective fracture reduction and fixation, reduce intraoperative blood loss, and significantly decrease soft tissue complications (18). Among these, the sinus tarsi incision has gained increasing clinical popularity due to a relatively low complication rate ranging from 3.6% to 6.3% (19). A retrospective study by Bremer et al (20) suggested that Sanders type II and III calcaneal fractures are optimal indications for fixation via the sinus tarsi approach. Other studies indicated that using a sinus tarsi incision in closed Sanders type II and III fractures could reduce soft tissue injury without compromising fracture reduction quality, thus accelerating fracture healing (21). Nevertheless, the sinus tarsi approach has limitations, such as poor fracture-site exposure, difficulties in achieving adequate fracture reduction, and potential injury to the sural nerve due to limited visibility (22). Shi et al (23) reported inferior restoration of the Böhler angle, posterior articular surface, and tuberosity alignment using the sinus tarsi approach compared with the extended lateral approach, indicating challenges in achieving high-quality reduction through this minimally invasive technique. Consequently, a meta-analysis involving numerous cases concluded that neither the L-shaped incision nor the sinus tarsi approach significantly reduced postoperative soft tissue complications (24).

With the advent of accelerated rehabilitation concepts, optimizing surgical strategies plays a decisive role in promoting early patient recovery. Studies suggest that timing significantly influences outcomes; performing surgery prematurely when swelling persists can result in insufficient skin healing and increased infection risk. Ideally, surgery should be performed within 3–5 days post-injury, especially when employing percutaneous or minimally invasive techniques (25). Forgon (26) reported excellent outcomes in 89% of 265 patients treated exclusively with minimally invasive percutaneous pin fixation. Magnan (27) described 54 cases treated with mini-external fixators for displaced intra-articular calcaneal fractures, achieving excellent-to-good outcomes in over 90% of patients. Percutaneous external fixation is considered suitable for all types of intra-articular calcaneal fractures, particularly those complicated by severe soft tissue injuries (28). In the current study, percutaneous minimally invasive reduction combined with crossbar external fixation was performed without waiting for swelling reduction. Although all three surgical methods evaluated demonstrated similar clinical outcomes for Sanders type II and III calcaneal fractures, the minimally invasive percutaneous technique offered additional benefits, including reduced surgical trauma, shorter hospital stays, and fewer complications. These advantages align with the contemporary trend towards minimally invasive treatments. Nevertheless, the selection of minimally invasive techniques should consider fracture type, soft tissue conditions, and surgeon expertise. For example, hindfoot fusion (Tibiotalocalcaneal Arthrodesis) can also be used as an alternative treatment option for some patients with poor soft tissue conditions, severe joint degeneration, or a combination of other complicating factors. The minimally invasive hindfoot fusion technique reported by Biz et al. (29), which utilizes retrograde intramedullary nailing to achieve stable fusion of the ankle joint to the talonavicular joint, has a low risk of infection and complications when managing cases with a high number of complications due to difficult and invasive reconstruction, with a low risk of infection and soft tissue injury. Combined with the clinical observations in this study, appropriate consideration of hindfoot fusion in some high-risk patients can further reduce the complication rate and improve the overall safety of surgery and patient satisfaction. Achieving optimal fracture reduction quality should never be compromised solely for minimally invasive benefits. Additionally, minimally invasive techniques require a learning curve and adequate surgical experience.

The results of this study showed that percutaneous minimally invasive combined with transverse rod external fixation technique has significant advantages in terms of shorter operative time, less bleeding, and lower complication rate, whereas the problem of higher wound complications (e.g., wound dehiscence, infection, and skin necrosis) of conventional open surgery, although it provides better recovery of radiographic indices (e.g., Böhler's angle), should not be ignored.

A study by Biz et al. (30) compared open internal fixation (ORIF) with percutaneous surgery (PS) in the treatment of calcaneal fractures. The results of this study showed that although the ORIF group was more favorable in restoring the Böhler angle, its complication rate (including wound healing problems and risk of infection) was significantly higher than that of the percutaneous surgery group.In contrast, the percutaneous minimally invasive technique used in this study effectively reduced soft tissue damage and related complications while ensuring good functional recovery.

This comparison not only further validates the advantages of percutaneous minimally invasive surgery in high-risk soft-tissue conditions, but also suggests that the choice of hindfoot fusion or other alternative surgical options may have some clinical value when targeting different patient groups, such as high-risk patients or those with poor soft-tissue conditions. In summary, in conjunction with the data from the study by Biz et al., we believe that more randomized controlled studies should be conducted in the future for different disease subtypes and patient conditions to optimize surgical strategies and improve overall treatment outcomes.

## 5 Conclusion

This study demonstrates that crossbar external fixation provides comparable anatomical and functional efficacy to conventional open reduction for Sanders type II/III calcaneal fractures; while exhibiting distinct advantages in reducing perioperative complications. The minimally invasive nature of this technique likely preserves critical vascular structures during fracture reduction, translating into lower iatrogenic risks without compromising long-term outcomes. Importantly, the approach enables more timely surgical intervention, addressing the clinical dilemma of delayed treatment associated with soft tissue management in traditional protocols. To advance this paradigm, future research should focus on three strategic domains: Biomechanical characterization of load-transfer mechanisms in percutaneous constructs to define long-term stability thresholds; Development of fracture pattern-specific selection criteria, particularly for highly comminuted subtypes where current techniques show limitations; Integration of intraoperative navigation technologies to achieve precision beyond conventional fluoroscopic guidance.

## 6 Limitations

There are still several limitations in this study:(i) The sample size is relatively small, and additional cases should be included in future studies; (ii) This study is a single-center retrospective analysis, which may introduce bias; thus, high-quality multi-center prospective studies are required to validate the findings;(iii) The follow-up duration is relatively short, necessitating further long-term follow-up assessments.

## Data Availability

The datasets presented in this study can be found in online repositories. The names of the repository/repositories and accession number(s) can be found in the article/Supplementary Material.
